# Correcting the bias against interdisciplinary research

**DOI:** 10.7554/eLife.02576

**Published:** 2014-04-01

**Authors:** Ehud Shapiro

**Affiliations:** Department of Computer Science and Applied Math and Department of Biological Chemistry, Weizmann Institute of Science, Rehovot, Israelehud.shapiro@weizmann.ac.il

**Keywords:** point of view, interdisciplinary research, scientific excellence, science policy, funding

## Abstract

When making decisions about funding and jobs the scientific community should recognise that most of the tools used to evaluate scientific excellence are biased in favour of established disciplines and against interdisciplinary research.

Many people have their abilities and achievements evaluated throughout their lives: this can be done via exam results for students, via salary and job title in the workplace, or via world rankings and earnings for individual sportsmen and women. Scientists too have their abilities and achievements scrutinised on a regular basis when they apply for jobs or promotion, or when they submit grant applications and manuscripts to funding agencies and journals. Identifying excellence is relatively straightforward in the highly structured worlds of, say, education or tennis. However, evaluating excellence in scientific research is difficult, and the scientific community is constantly struggling to improve the ways it measures and rewards excellence.

Scientists who leave the safe haven of their home discipline to explore the uncharted territory that lies outside and between established disciplines are often punished rather than rewarded for following their scientific curiosity.

A case in point is evaluating excellence in interdisciplinary research. By that I specifically mean research that aims to integrate two (or more) existing disciplines. My own research involves applying various tools and methods from computer science to problems in biology. However, the views expressed here apply equally to, say, physicists thinking about gene expression, mathematicians modelling bacterial colonies, and to any other research that lies at the perimeters of the well-established disciplines. The difficulties associated with recognising excellence in interdisciplinary research have an unfortunate side effect: scientists who leave the safe haven of their home discipline to explore the uncharted territory that lies outside and between established disciplines are often punished rather than rewarded for following their scientific curiosity.

## Is there really a bias against interdisciplinary research?

One objective measure of the bias against interdisciplinary research is provided by the success rates of researchers who apply to the European Research Council (ERC) for ERC Advanced Grants and ERC Starting Grants. Although excellence is the sole selection criterion used to award these grants, the success rate for applications categorised as interdisciplinary has been lower than the success rate for single-discipline applications in each of the first four years of these schemes (Labastida, 2013).

Such objective measures of the bias against interdisciplinary research are very difficult to come by, yet subjective and/or personal experiences are commonplace. It seems a common occurrence for research outside of the well-established disciplines to be rejected outright by journal editors as uninteresting to the community. Furthermore, even if a submitted manuscript passes the initial screen, the editor may have difficulties finding referees who have the expertise needed to review the paper.

And when it comes to nourishing particular disciplines, those in positions of power—such as department heads in universities or programme managers at funding agencies—face the daily grind of a zero-sum struggle for resources. This means that they are often only able or willing to support interdisciplinary research with lab space or research grants if doing so does not erode the resources available to researchers working in that particular discipline. This inevitably tips the scales against researchers who do not fit comfortably into any existing discipline.

## How and why does the bias arise?

Most researchers work in what could be termed ‘dense scientific communities’. By this I mean well-established scientific communities in which the different members attend the same meetings, publish in the same journals, cite each other’s papers and often collaborate and compete with each other. In such dense scientific communities, the goals of the field are well defined and well known, and are addressed simultaneously by many scientists in a competitive way, much like an Olympic race. Moreover, in such communities the preferred venues for the publication of new results—high-impact journals in some communities, competitive conferences in others—are also widely recognised and community members are more than willing to review manuscripts written by their colleagues, because it helps them to stay on top of the competition and to be inspired by yet-unpublished research. Publications in such communities also get cited quite rapidly and in large numbers, by the many peers working in the same area.

In dense scientific communities, the ‘elders’ are well recognised and have the authority to set the rules by which excellence in this community is measured. These elders are also forthcoming with letters of recommendation for their best intellectual offspring. Young researchers who play by the rules and successfully address problems that have been deemed important by the elders are rewarded handsomely and, over time, these young researchers become elders themselves, thus ensuring that the community continues to flourish.

Often the best that one can hope for is to recruit unsuspecting students as company for the journey.

This situation is in stark contrast to that of researchers operating at and between the boundaries of established disciplines. Genuine interdisciplinary research is nothing like a competitive race. It is much more like a solitary exploratory hike through an uncharted landscape. There are no community elders to give guidance, to define and rank the important research goals, or to write recommendation letters to their intellectual offspring. There are no peers to compete with or use as reviewers; and there are no community-specific journals or conferences. In general, the intricate and encompassing community support structure present in dense scientific communities is completely missing. Often the best that one can hope for is to recruit unsuspecting students as company for the journey.

All this means that many of the so-called ‘objective measures’ that are used to assess researchers—such as impact factors (with all their flaws), citation counts, or letters of recommendation—are highly biased in favour of researchers who belong to dense scientific communities at the expense of those who are members of sparse scientific communities.

## Why should we care and what can we do?

Although recognising excellence at and between the boundaries of established disciplines is difficult, it is essential that we keep trying to identify scientists who have the potential to make real breakthroughs in these areas. If these scientists are not identified and funded, then—almost by definition—certain highly promising interdisciplinary areas might remained unexplored because no one else is working in them. This is not a problem in dense scientific communities: if we fail to recognise and support the very best person working towards a particular important goal in this field, then someone else will probably reach the same goal, albeit with some delay. Unfair as it may be, the progress of science is relatively unaffected by such an outcome. Hence, as rare as excellence may be in the outback of interdisciplinary research, it should be actively sought, identified and rewarded even more than excellence in dense communities.

How can we ensure that excellence in areas other than established disciplines is actively sought, identified and rewarded even more than excellence in dense scientific communities? First we must recognise that the three tools that are most widely used to evaluate researchers—measures based on impact factors, citation counts and recommendation letters—are highly biased in favour of members in dense communities. Once we do, the next step should be to actively correct this bias.

For example, one code-word used for rejecting interdisciplinary grant proposals is ‘over-ambitious’. This often stands for saying that the scientist intends to do something that is radically different from what has been done until now and does not follow the ‘rules’ of the reviewer’s home scientific community. Perhaps such judgments should be banned. What is wrong with being over-ambitious? Besides, the predictability of a scientific endeavour is arguably inversely proportional to how innovative it is. So maybe any scientific project that is not ‘over-ambitious’ is by definition not innovative enough?

A useful countermeasure would be to develop an objective measure of the density of various scientific communities that could be taken into account when making decisions about funding and jobs. If two scientists are judged equal on the basis of the standard measures, then the scientist from the less dense community (see figure) could be ranked higher to compensate for the biases against researchers in these areas.

**Figure.**
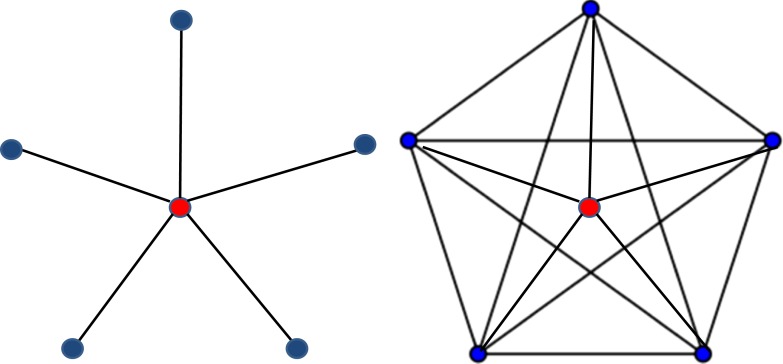
Sparse and dense scientific networks. In a scientific social network, what matters most are citation relations. The social network of a particular scientist, say Mary Smith (red circle), consists of all the scientists (blue circles) cited by Mary or citing Mary, with edges connecting those scientists who have cited each other. The resulting network will lie somewhere between two extremes: a maximally dense network (right), in which all the scientists in the network cite each other, and a maximally sparse network (left) in which there are no citations among the scientists except citations by or to Mary.

More sophisticated analysis could also be applied. For example, it would be useful to identify dense scientific communities that receive very little input from, and exert very little influence over, other fields of science; decisions to fund such ‘mutual-admiration societies’ should be subject to higher than usual levels of scrutiny. Conversely, scientists who have integrated two previously unrelated areas of research to open up completely new fields of inquiry could also be identified by such an analysis, and recognised accordingly.

More research should be devoted to measuring the density of scientific communities (probably based on various techniques from graph theory), and more thought should be given as to how this measure can be factored into the evaluation of scientific excellence. Let me be clear, however: any such correction is not intended to compensate an interdisciplinary scientist for his or her personal hardships. Rather, such a correction is needed to prevent seemingly objective evaluation tools from misleading us and, as a result, holding back scientific progress by diverting funds and jobs from people who have the potential to perform truly ground-breaking science.
